# Energetically Favored 2D to 3D Transition: Why Silicene Cannot Be Grown on Ag(111)

**DOI:** 10.1021/acs.nanolett.4c00140

**Published:** 2024-03-19

**Authors:** Chi-Ruei Pan, Mei-Yin Chou

**Affiliations:** † School of Physics, Georgia Institute of Technology, Atlanta, Georgia 30332, United States; ‡ Institute of Atomic and Molecular Sciences, Academia Sinica, Taipei 11529, Taiwan; § Department of Physics, National Taiwan University, Taipei 10617, Taiwan

**Keywords:** Silicene, 2D film growth, 2D to 3D transition, dewetting phenomenon, Volmer−Weber growth mode

## Abstract

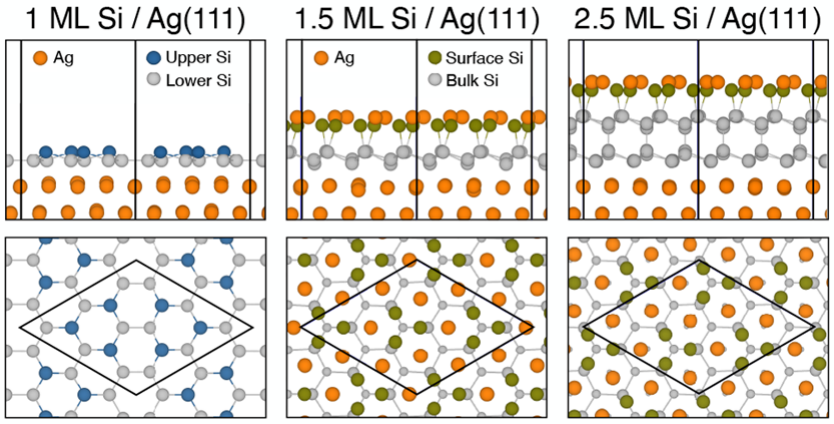

Silicene, a two-dimensional (2D) Si monolayer with properties similar to those of graphene, has attracted considerable attention because of its compatibility with existing technology. Most growth efforts to date have focused on the Ag(111) substrate, with a 3 × 3 phase widely reported below one monolayer (ML). As the coverage increases, a √3 × √3 pattern frequently emerges, which has been proposed by various experimental investigations as a  reconstructed structure. We report first-principles calculations to understand this series of observations. A major finding from our energetics studies is that Si growth on Ag(111) beyond one ML will switch to the Volmer–Weber mode, forming three-dimensional sp^3^ films. Combining with the condition that the 3 × 3 monolayer on Ag(111) does not have the correct buckling pattern of freestanding silicene, we conclude that silicene cannot be grown on Ag(111) and that a 2D to 3D transition is energetically favored beyond one ML.

Following the successful synthesis of graphene, researchers have explored other potential candidates for a two-dimensional (2D) honeycomb lattice based on group-IV elements. The fabrication of silicene, a 2D Si monolayer, has stimulated tremendous interest and efforts because its compatibility with existing electronic technology may be advantageous, even though no 2D structure of Si has ever been found in nature. It was theoretically predicted that freestanding silicene is thermally stable in the form of a slightly buckled honeycomb lattice^1^ and that massless Dirac Fermions also exist in this freestanding structure.^2^ The buckling in the freestanding silicene monolayer was found to create a hybridization feature of distorted sp^3^ instead of distorted sp^2^.^3^ Silicon does not have three-dimensional (3D) allotropes like layer-structured graphite in nature, but it has a stronger spin–orbit coupling than C, making silicene a potential candidate for the quantum spin Hall effect^4^ and the quantum anomalous Hall effect.^5^

The most frequently used and widely studied substrate for the growth of silicene is Ag(111). A variety of structures, including 3 × 3, , and  (with respect to the 1 × 1 unit cell of silicene), have been reported depending on the growth temperature and Si coverage.^6−8^ In addition, a common evolution of the structure as a function of temperature and deposition time has been reported.^9−11^ With Si coverage below one monolayer (ML), a 3 × 3 phase was discovered and well studied. Its atomic structure was determined as a lattice-matched monolayer structure on a 4 × 4 Ag(111) unit cell. However, due to the Si–Ag interaction, this phase has an irregular buckling pattern compared with freestanding silicene, resulting in a flower-like pattern observed by scanning tunneling microscopy (STM). When Si atoms are deposited beyond one ML, another phase with a √3 × √3 pattern was usually observed in STM. In this phase, Chen et al.^12^ found a linear energy dispersion based on the quasiparticle interference pattern through 2D real-space mapping. However, by adding more data points near the Fermi level, Arafune et al.^13^ demonstrated that the dispersion is parabolic, although it becomes more linear upon moving away from the Fermi level. At a temperature lower than 40 K, Chen et al.^14^ also found two similar √3 × √3 patterns with reduced symmetry and different orientations. These peculiar properties motivated us to study the √3 × √3 phase in further detail.

To date, numerous experimental studies have reported that as the temperature of the Ag substrate is increased above 500 K or the Si coverage exceeds one ML, a √3 × √3 reconstruction appears on the terrace of the multilayer region.^15−23^ However, its structure and stability are still not fully resolved. Chen et al.^24^ proposed a model for the √3 × √3 pattern with one-third of Si atoms highly upward-bucked, while Cahangirov et al.^25^ assumed additional adsorbed Si atoms on silicene to form dumbbell structures. Recent experimental results unveiled that the surface of this phase is likely to be a  reconstructed structure, the honeycomb-chain-trimer (HCT) model of the Si(111) surface with adsorbed Ag atoms.^18,20,22,23^ In particular, comparing the STM topology of Ag on Si(111) with that of Si on Ag(111) beyond one monolayer, a similar √3 × √3 symmetry was found.^19^ Another question of interest is whether the bonding environment of the underlying Si beyond one monolayer is diamond-like or graphite-like. Vogt et al.^17^ showed that the heights from layer to layer in the √3 × √3 phase formed in the multilayer Si region are close to integer multiples of 0.31 nm, which corresponds to the thickness of one double-layer of the Si bulk in the [111] direction. De Padova et al.^21^ performed accurate measurements for the lattice constant and the Raman intensity, and they showed that at a higher growth temperature (∼300 °C), no significant difference was found between the √3 × √3 “multilayer silicene” and . Thus, “multilayer silicene” is believed to be essentially the Si bulk covered by Ag atoms with a HCT √3 × √3 reconstruction. Interestingly, Kawakami et al.^26^ reported an anomalous dewetting phenomenon followed by further deposition of Si after the completion of the first ML with the reappearance of the pristine Ag(111) surface. To understand these experimental observations and to determine the atomic configurations and resulting electronic properties, a first-principles study is indispensable.

In order to understand the growth mechanism for the √3 × √3 phase, we studied the atomic structure and the electronic properties of the HCT structure on multilayer Si (with the Ag substrate) and made comparisons with measurements for the √3 × √3 phase. In addition, we report detailed calculations of the energetics for relevant configurations in order to identify the most favorable structure when more than one ML of Si is deposited on Ag(111). Starting from the 3 × 3 ML phase on the Ag substrate, we construct the structure of the √3 × √3 phase with a Si coverage of 1.5 2.5, 3.5 ML, and investigate their electronic properties and energetics. We present the coverage-dependent average energy per Si atom in different configurations and conclude that the 3D Volmer–Weber mode, not the Stranski–Krastanov mode is energetically favorable beyond one ML. Since the 3 × 3 ML phase does not have the desirable buckling of silicene, and with more coverage the film structure turns into sp^3^, our computational results indicate that it is highly unlikely to grow silicene ML or silicene multilayers on Ag(111).

As a reference, we have calculated the relaxed structure of the HCT model for , as illustrated in Figure 1. This structure can be understood as follows. A cut of the Si(111) surface through the vertical covalent bonds results in an unstable surface layer with three dangling bonds on each Si atom (dark green spheres in Figure 1). With additional Ag atoms (orange spheres) on the surface, three Si atoms move closer to each other, saturating two of the dangling bonds. Each Si atom also binds more strongly with one of the nearest Ag atoms, pulling it slightly away from the center of the hexagon (Figure 1b). The nearest-neighbor surface Ag atoms form equilateral triangles with two alternating orientations connected at the vertices and appearing as a honeycomb chain-like structure. Therefore, the name HCT is given to this model.

**Figure 1:**
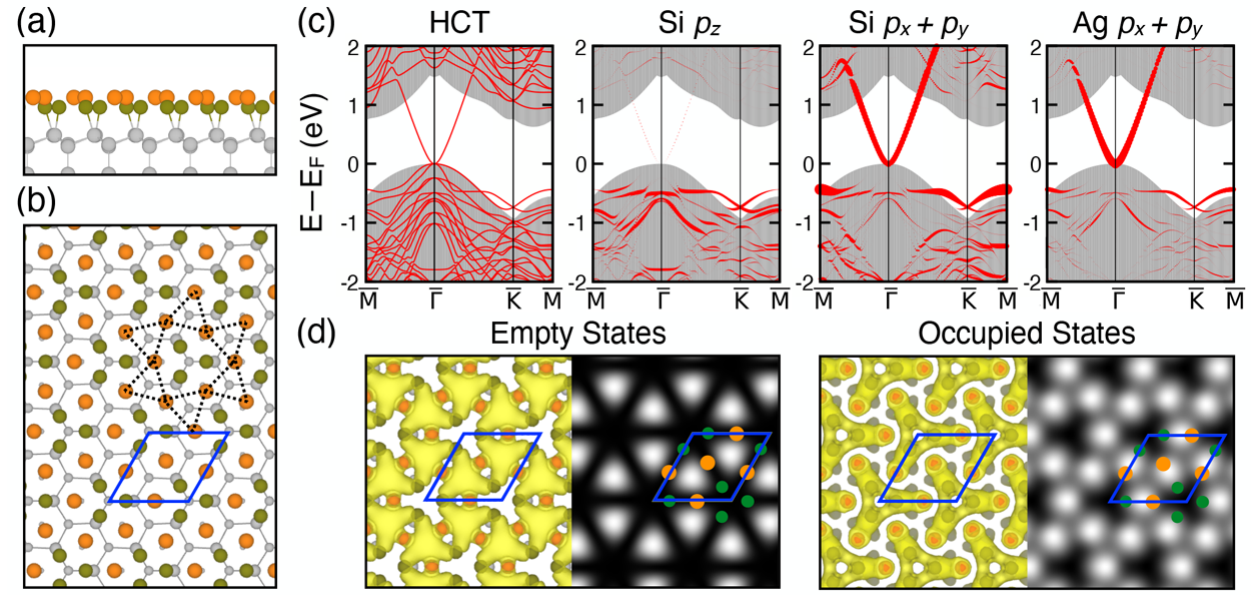
(a) Side view and (b) top view of the honeycomb-chain-trimer (HCT) model of . The orange, dark green, and gray spheres correspond to surface Ag, surface Si, and lower-layer Si atoms, respectively. The blue rhombus in (b) shows a  unit cell. (c) Electronic band structure of the HCT model and projected bands for different orbitals of surface Si and Ag atoms. The background gray region represents the Si bulk-projected bands. The radii of the red circles are proportional to contributions from each state. The valence band maximum (VBM) of Si is set at zero. (d) Isosurfaces of the charge density distribution and simulated STM images for the empty states (within 0.5 eV above the Fermi level) and the occupied states (within 1 eV below the Fermi level), respectively. The isosurface level is 0.0003 (0.004) *e*/*a*_0_^3^ for the empty (occupied) states, where *a*_0_ is the Bohr radius. The STM simulation is obtained using the constant-height mode with a tip height of 2.5 Å by the p4vasp program (https://github.com/orest-d/p4vasp).

The calculated band structure of the HCT model exhibits a surface band lying in the band gap of the Si bulk with a nearly linear dispersion in the energy range from 0.1 to 1 eV, as shown in Figure 1c. The projected band structure indicates that the almost linear dispersion is dominated by the *p*_*x*_ + *p*_*y*_ orbitals of Si and Ag, indicating their mutual interaction. In contrast, the linear bands around the Fermi level in freestanding silicene are predicted to be the *p*_*z*_ orbital of Si. A calculated group velocity of approximately 0.86 × 10^6^ m/s is found for the linear dispersion in the Γ̅ K*®* direction, which is consistent with the experimental value of (1.2 ± 0.1) × 10^6^ m/s derived from the quasiparticle interference pattern for the Si film grown on Ag(111).^12^ The charge density isosurfaces for the empty and occupied states are shown in Figure 1d. We note that for the empty states, the protrusion of the charge occurs at the center of the Ag trimers, resulting in the bright spots observed in simulated STM images. In contrast, occupied states represent bonding between the nearest Si and Ag on the surface.

When the Si coverage is lower than one ML on Ag(111), a unique flower-like pattern can be observed through STM. It can be described by a 3 × 3 Si supercell (SC) lattice-matched with a 4 × 4 SC of Ag(111). This 3 × 3 phase has been thoroughly examined,^27−30^ and its atomic structure, shown in Figure 2a, has been identified as a honeycomb silicene lattice with a distinct buckling pattern. This distorted structure is caused by the significant interaction between Si and the Ag substrate, breaking the regular buckling symmetry of freestanding silicene and losing the preferred linear dispersion of 2D massless Dirac electrons. Theoretical calculations have revealed that the *p*_*z*_ states shift downward.^31^ This feature can explain the absence of the Landau-level sequences in scanning tunneling spectroscopy (STS) studies^32^ and the failure of finding Dirac cones in ARPES measurements.^33^

**Figure 2:**
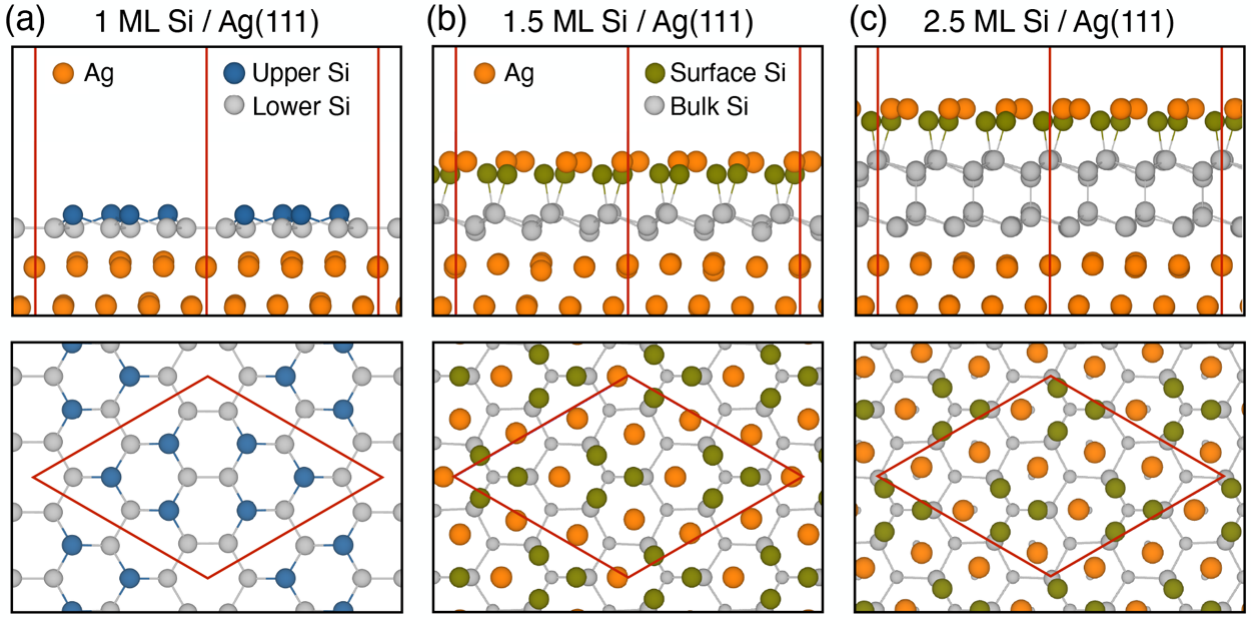
Side view (upper panel) and top view (lower panel) of the relaxed atomic structure of (a) one ML of Si on Ag(111) (3 × 3 phase), (b) 1.5 ML of Si on Ag(111) (1.5 ML HCT), and (c) 2.5 ML of Si on Ag(111) (2.5 ML HCT), in which the orange, dark green, and gray spheres correspond to Ag, surface Si, and lower-layer Si atoms, respectively. The red rhombuses show 3 × 3 unit cells used in the calculation. In a, the dark blue spheres denote upward buckled Si atoms. For all the top views, Ag substrates are not shown for simplicity.

After the Si coverage is beyond one ML, the √3 × √3 pattern emerges as another stable phase and continues to show up on the terrace of each multilayer step. In order to study the effects of the Ag substrate and the growth mechanism, we investigated the few-layer regime in this study. First, we build the HCT model directly on top of the 1 ML 3 × 3 phase by adding 9 Si atoms (0.5 ML) and 9 Ag atoms (0.5 ML) above it in a SC. After relaxation, the optimized configuration is illustrated in Figure 2b, which has almost the same surface structure as that of the HCT model. This model is then named the “1.5 ML HCT”. Note that the bottom-layer Si restores the nearly symmetric regular-buckled pattern in bulk Si and that the additional 0.5 ML of Si can form covalent bonds with the first ML below with their bonds slightly tilted away from the vertical lines. However, the average Si energy per atom in this structure is higher than that of the 3 × 3 phase (to be discussed later), and the projected band structure (Figure 3a) shows that the nearly linear dispersion of the HCT model is distorted and strongly modified by the Ag substrate. Therefore, we next add another ML Si to the “1.5 ML HCT” configuration to form the “2.5 ML HCT” model, shown in Figure 2c, where the surface also has the HCT structure after relaxation. In contrast, a similar nearly linear dispersion with a large group velocity originating from the HCT model near the Γ̅ point can be found, as labeled by the blue dashed lines in Figure 3b.

**Figure 3:**
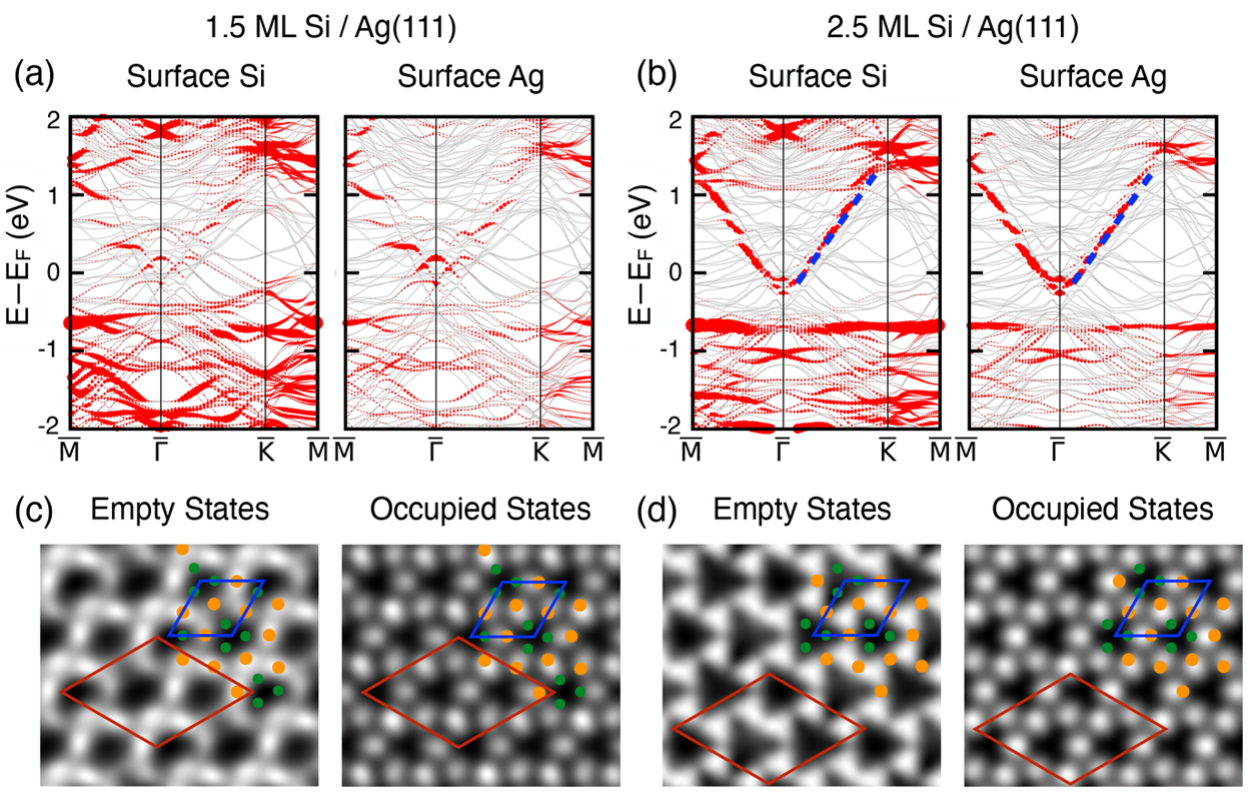
Projected band structure on the *p*_*x*_+*p*_*y*_ orbitals for the (a) 1.5 ML HCT and (b) 2.5 ML HCT models. The radii of the red circles are proportional to the contributions from each state. The Fermi level was set at zero. The blue dashed lines show the nearly linear dispersion in some parts of the energy bands. The STM simulated images for (c) 1.5 ML HCT and (d) 2.5 ML HCT are carried out in the same manner as used to produce Figure 1d. Empty states (occupied states) are chosen from within 0.5 eV (1.0 eV) above (below) the Fermi level. The STM simulation is done with the constant-height mode with a tip height of 2.5 Å. The blue (red) rhombuses show √3 × √3 (3 × 3) unit cells with respect to 1 × 1 Si(111).

We also performed the STM simulation for the “1.5 ML HCT” and “2.5 ML HCT” models. Results are shown in Figure 3c and d, respectively. While occupied-state images are all similar in showing the bonding between the surface Si and Ag, their empty-state images are quite different. After the coverage is above 2.5 ML, the empty-state image starts to show a close resemblance to the HCT model (six bright spots with a rotational symmetry of 120°), which is also consistent with the appearance of the HCT surface band in the “2.5 ML HCT”.

Recently, a dewetting phenomenon was reported for Si growth on Ag(111).^26^ At the beginning of the growth, Si forms the 3 × 3 phase that “wets” the surface to extend its coverage. As the deposition time increases and the coverage goes beyond one ML, the √3 × √3 phase starts to emerge, accompanied by the diminishment of the 3 × 3 phase and the reappearance of the pristine Ag substrate that was initially covered. These interesting observations motivate us to further investigate the energetics from our first-principles calculations in order to explain this growth behavior.

We consider the energy competition of various configurations, as shown in Figure 4a. Structures I, II, III, and IV in the upper panels contain the 1 ML 3 × 3 phase (blue) that completely covers the Ag(111) surface (orange). Additional Si atoms form the HCT √3 × √3 phase with different thicknesses. The ideally buckled Si layers as in the bulk are shown in gray, and the 0.5 ML surface Si is shown in green (see the structures in Figure 2). For the sake of simplicity, surface Ag atoms in the HCT phase are not marked. In comparison, structures I′, II′, III′, and IV′on the lower panels in Figure 4a are configurations exhibiting the “dewetting” phenomenon with the presence of pristine Ag(111) and multilayer Si in the HCT √3 × √3 phase, without 1 ML 3 × 3 phase. To study the energetics, we evaluate the binding energy of Si by taking the difference between (1) the energy of the configuration under consideration containing Si and Ag atoms and (2) the energy sum of the clean Ag surface and the same number of free Si atoms. The edge energy is neglected, since its contribution has a relative scale of 1/√*N*, where *N* is the large number of unit cells in the 2D Si film. Since structures I′, II′, III′, and IV′contain only one specific pattern for the Si layers, the change of the Si coverage will only modify the area of the clean Ag surface, whose energy is in the energy reference by definition. Therefore, the resulting average binding energy per Si atom will not change with the Si coverage for configurations I′, II′, III′, and IV′. In contrast, for a given Si coverage in structures I, II, III, and IV, we need to first determine the relative fraction for each phase present. Then we evaluate the total energy of the system by adding these portions. Since the HCT √3 × √3 phase has additional surface Ag atoms, additional bulk Ag atoms are added to the substrate when needed in order to keep the number of Ag atoms the same in all total energy calculations.

**Figure 4:**
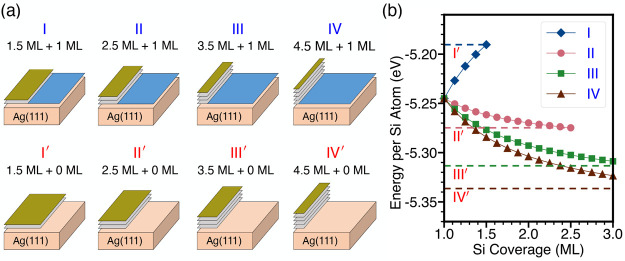
(a) Schematics of possible structures for a Si coverage larger than one ML deposited on Ag(111) with different combinations of 1 ML 3 × 3, 1.5 ML HCT, 2.5 ML HCT, and beyond. (b) The calculated average energy per Si atom (see main text) for the structures in the upper panels of (a) as a function of Si coverage presented by the solid symbols. The average energy for pure 1 ML 3 × 3, 1.5 ML HCT, 2.5 ML HCT, 3.5 ML HCT, 4.5 ML HCT are labeled by the dashed lines for reference, respectively.

The calculated average energies per Si atom for the configurations in Figure 4a are plotted in Figure 4b as a function of the Si coverage. Results for “wetted” structures I, II, III, and IV are shown by solid symbols, while the energies for the “dewetted” structures, I′, II′, III′, and IV′, with only the pure HCT structure, are labeled by constant dashed lines. These constant lines move to lower energies as the thickness increases, with the limit being the bulk energy of −5.417 eV. At one ML, the energy of the “wetted” 3 × 3 phase is −5.245 eV/atom, higher than that of structures II′, III′, and IV′, indicating that a 3D structure is energetically more favorable, while the 2D 3 × 3 phase is a metastable phase that appears in certain growth conditions. As the coverage increases beyond one ML, the energy of the structure with a 1.5 ML component (structure I, blue diamond line in Figure 4b) goes up. In comparison, the “2.5 ML HCT” √3 × √3 phase in Figure 2c and configurations with even thicker layers are energetically more favorable. Therefore, the intermediate 1.5 ML structure acts as an energy barrier in transforming to more stable 3D configurations. This explains the observation that the system tends to complete the first-layer growth of the metastable 2D 3 × 3 phase, as observed in time-recorded deposition experiments on the Ag substrate.^5,9^

To be specific, after completion of the first layer, additional Si atoms have no access to the Ag substrate and can only interact with the first ML 3 × 3 phase. The system is forced to overcome the energy barrier by transforming to the intermediate state (Structure I) and can soon lower its energy by forming a “2.5 ML HCT” and beyond. Interestingly, the energy results in Figure 4b indicate that structures with the single-layer 3 × 3 phase still present (structures II, III, and IV) have a higher energy than those without the single-layer 3 × 3 phase (structures II′, III′, and IV′). For example, the energy of structure II is higher than that of structure II′, and the energy of structure III is higher than that of structure III′, etc.

These energetics results indicate a growth mode change as the Si deposition time increases. After finishing the first ML of the 3 × 3 phase, the Si growth changes to the Volmer–Weber mode, in which the Si interaction with the HCT island is stronger than that with the Ag substrate. As a consequence, the formation of a 3D island is preferred, with the reappearance of the pristine Ag surface. Therefore, our calculated energetics results successfully explain the experimental finding that the first-layer 3 × 3 phase starts to diminish as the 3D Si islands emerge; namely, the observed dewetting phenomenon is energetically driven. Our results also indicate that the minimal thickness of the Si thin film with the stable √3 × √3 phase would be 2.5 ML. Once this 2.5 ML film is formed, a cascade toward thicker HCT structures can be expected.

In summary, we have performed first-principles calculations for various configurations of Si multilayers on Ag(111) and studied their energetics and electronic structures. Below one monolayer coverage, we find that the 3 × 3 phase is a metastable structure with a specific buckling pattern that does not provide the desirable linear dispersion as in freestanding silicene. When the coverage is beyond one monolayer, the three-dimensional Si bulk structure is energetically favorable, with the surface exhibiting a √3 × √3 phase consistent with the reconstructed honeycomb-chain-trimer model in . The electronic structure of the √3 × √3 phase with multilayer Si is also found to be similar to that of this reconstructed configuration for the Si surface. Our calculated results indicate that upon finishing the first monolayer, the Si growth changes to the Volmer–Weber mode with three-dimensional Si sp^3^ structures and the reappearance of pristine Ag(111), instead of continuing with the Stranski–Krastanov mode. This finding is consistent with the dewetting phenomenon observed in the time-resolved Si deposition experiments on Ag(111). This energetically favored transition from two- to three-dimensions prevents the successful growth of desirable silicene systems on Ag(111).

## Computational Details

We have performed first-principles calculations within density functional theory (DFT) as implemented in the Vienna Ab initio Simulation Package (VASP).^34,35^ The plane-wave basis set was adopted, and the pseudopotential was approximated by the projector augmented wave (PAW) method.^36^ The Perdew–Burke–Ernzerhof (PBE) form^37^ of the exchange-correlation functional was used in this calculation. We used a periodic slab with a vacuum region of about 11–15 Å. For the HCT model, the slab contained ten Si layers as the substrate with the bottom eight layers of Si fixed during relaxation. For the “1.5 ML HCT” and “2.5 ML HCT” models, the slab contained six layers of Ag with the bottom two layers fixed. The energy cutoff of the plane-wave basis set was 500 eV (300 eV), and the Monkhorst–Pack k-point mesh of 7 × 7 × 1 (5 × 5 × 1) was used for the HCT model (HCT model on the Ag substrate). The structure was fully relaxed until each atom’s force was less than 0.01 eV/Å.

## Notes

A previous experimental study^38^ using low-energy electron microscopy to image the growth of silicene layers on Ag(111) also observed the reappearance of the clean Ag(111) surface when the surface was almost covered by monolayer silicene, indicating a possible “sp2-like” to “sp3-like” phase transition of Si hybridization beyond one monolayer.
